# chaste codegen: automatic CellML to C++ code generation with fixes for singularities and automatically generated Jacobians

**DOI:** 10.12688/wellcomeopenres.17206.1

**Published:** 2021-10-12

**Authors:** Maurice Hendrix, Michael Clerx, Asif U Tamuri, Sarah M Keating, Ross H Johnstone, Jonathan Cooper, Gary R Mirams

**Affiliations:** 1 Centre for Mathematical Medicine & Biology, University of Nottingham, Nottingham, UK; 2 Digital Research Service, Information Sciences,, University of Nottingham, Nottingham, NG8 1BB, UK; 3 Centre for Advanced Research Computing, University College London, London, WC1E 6BT, UK; 4 Computational Biology & Healthcare Informatics, Department of Computer Science, University of Oxford, Oxford, OX1 3QD, UK

**Keywords:** CellML, cardiac electrophysiology, code generation, C++, jacobian, singularity, GHK equation

## Abstract

Hundreds of different mathematical models have been proposed for describing electrophysiology of various cell types. These models are quite complex (nonlinear systems of typically tens of ODEs and sometimes hundreds of parameters) and software packages such as the Cancer, Heart and Soft Tissue Environment (Chaste) C++ library have been designed to run simulations with these models in isolation or coupled to form a tissue simulation. The complexity of many of these models makes sharing and translating them to new simulation environments difficult. CellML is an XML format that offers a solution to this problem and has been widely-adopted. This paper specifically describes the capabilities of chaste_codegen, a Python-based CellML to C++ converter based on the new cellmlmanip Python library for reading and manipulating CellML models. While chaste_codegen is a Python 3 redevelopment of a previous Python 2 tool (called PyCML) it has some additional new features that this paper describes. Most notably, chaste_codegen has the ability to generate analytic Jacobians without the use of proprietary software, and also to find singularities occurring in equations and automatically generate and apply linear approximations to prevent numerical problems at these points.

## Introduction

Within the area of electrophysiology, there are hundreds of models describing biological behaviour. Many of these models are complex systems of tens of ordinary differential equations (ODEs) with hundreds of parameters, making translation into different simulation software time-consuming and prone to transcription errors. This also makes sharing models between different tools and application areas difficult. CellML ^1^ addresses this problem by offering a way to describe mathematical models in an XML-based format, independent of the choice of programming language or tools used to simulate or analyse the models. It was originally created with the Physiome Project in mind, and a large repository of well over a hundred CellML electrophysiology models is available on the Physiome Model Repository ^2, 3^ (PMR, https://models.physiomeproject.org/cellml). CellML sees continued development by the research user base ^4^ and several tools are available to support modelling using CellML models.

CellML models can be imported into various simulation tools such as the Cancer Heart and Soft Tissue Environment (Chaste) ^5^, OpenCOR ^6^, Myokit ^7^, and model comparison tools such as the Cardiac Electrophysiology Web Lab ^8, 9^. This paper describes the development of chaste_codegen — a Python 3 CellML code generator which is now used by Chaste. Its functionality is largely inspired by PyCML ^10^, a Python 2 implementation of CellML model import into Chaste. However chaste_codegen is an all-new implementation which includes a number of new features previously unavailable to the community, as described below.

## Methods

### Implementation

chaste_codegen builds on the cellmlmanip library and Jinja2 ^11^ templates to generate Chaste C++ code from CellML files. It is available as a standalone command-line tool for Python 3 on Windows Linux and Mac, and has been integrated into the latest release of Chaste.

CellML is a model definition language and as such it describes the model and its equations, but does not describe how the the equations should be solved or how experiments are run. cellmlmanip is a new Python 3 library for parsing CellML into ordered equations and metadata. It is a flexible component that can read CellML and enable it to be used for a variety of purposes, such as translating CellML models into other formats, or into code for various simulation packages. Separation of the parser and simulator has the advantage of creating a resource for the CellML community, which will ultimately be much easier to maintain than writing a bespoke CellML parser for each application. Key features of cellmlmanip are: using SymPy to represent mathematics (with the full manipulation capabilities thereof available), tracking physical units and performing conversions as needed, and managing and querying RDF metadata annotations on models. Cellmlmanip currently supports CellML version no 1.0, but it could easily be adapted to support CellML version no 2.0 ^4^. However there are currently no plans to do this, because of the ongoing development of libcellml ^4^. libcellml is a more general propose CellMl 2.0 library should offer the ability to not only read and manipluate CellMl 2.0 models but also write adjusted models back again.

SymPy is a python library for symbolic mathematics ^12^ that offers a number of convenient features for chaste_codegen. Most notably, it provides us with the ability to calculate Jacobians algebraically, to recognise patterns, rewrite equations, and to extract common terms in a set of equations. It also comes with a convenient printing mechanism, separating the mathematics from their representation.

Jinja2 is a templating language for Python, modelled after Django’s templates. Using Jinja2 allows us to separate the logic from the code output, which allows generating code for a number of different solvers, as described in detail in Cooper *et al.* ^13^. Jinja2 templates should allow easy adaptation to export code in other programming languages. chaste_codegen and its stack of dependencies are all free and open source. 

### Analytic Jacobians

Within Chaste one of the main solver types available is CVODE, which performs well for the stiff systems that feature in many electrophysiology models. CVODE can be sped up if the user provides a method to return the Jacobian matrix for the ODE system (with entries defined as the partial derivative of each ODE’s right-hand-side with respect to each state variable). If this is missing CVODE derives an approximation for it based on finite differences ^14^. PyCML required a Jacobian matrix to be pre-computed by the proprietary Maple software. In chaste_codegen we have integrated calculation of a Jacobian matrix at runtime, by making use of the SymPy library.

We contrasted previously existing code, using externally created Jacobians, with the newly generated models with automatically generated Jacobians. The validity of the generated Jacobians was assessed by comparing numerical results of runs of a number of different models.

### Singularities

Many electrophysiology models use a formulation for ion currents based on the Goldman-Hodgkin-Katz (GHK) flux equation ^15^, or feature equations with similar structure. Unfortunately such equations can introduce *singularities* into a model. Here, we define singularities as situations where an equation tends to 0 */*0 close to a particular value of a model variable (usually membrane voltage, *V*).

In general terms, these GHK-style equations take the form:

$$\;C(V)=f(V)\frac{\left(V-v_{0}\right)}{e^{B(V-v_{0})}-1},\;(1)$$

where *B* and *v*_0_ are constants, and *f* ( *V*) is any function of *V* (that may also be simply a constant) and *v*_0_ is the voltage at which we hit the singularity. As per Johnstone ^16^, we can simplify this notation by defining

$$\;A(V)=\frac{f(V)}{B},\;(2)$$

and use the substitution

$$\;U=B\times (V-v_{0}),\;(3)$$

to leave Equation (1) with a nondimensional fraction term that encapsulates the singularity,

$$\;C(V)=A(V)\frac{U}{e^{U}-1}.\;(4)$$

For convenience we also define

$$\;g(U)=\frac{U}{e^{U}-1}.\;(5)$$

We can now see that at as *U* → 0 there is a singularity as both the top and bottom of the fraction in Equation (5) tend to zero ( *e ^U^* → 1, so *e ^U^* – 1 tends to zero).

It is important to point out that mathematically the value of the equation remains well defined; no physics in the model is breaking down as we get close to 0 */*0. That is, there is a finite value to which the expression evaluates which we will tend towards as we get closer to the singularity (a *limit*), and this can be found analytically using approaches such as L’Hôpital’s rule, as we will discuss later. But when evaluating such equations numerically, it is possible to reach voltages close to (or at) the singularity where the numerical evaluation is not just inaccurate but often tends to ±∞.

**Figure 1.**
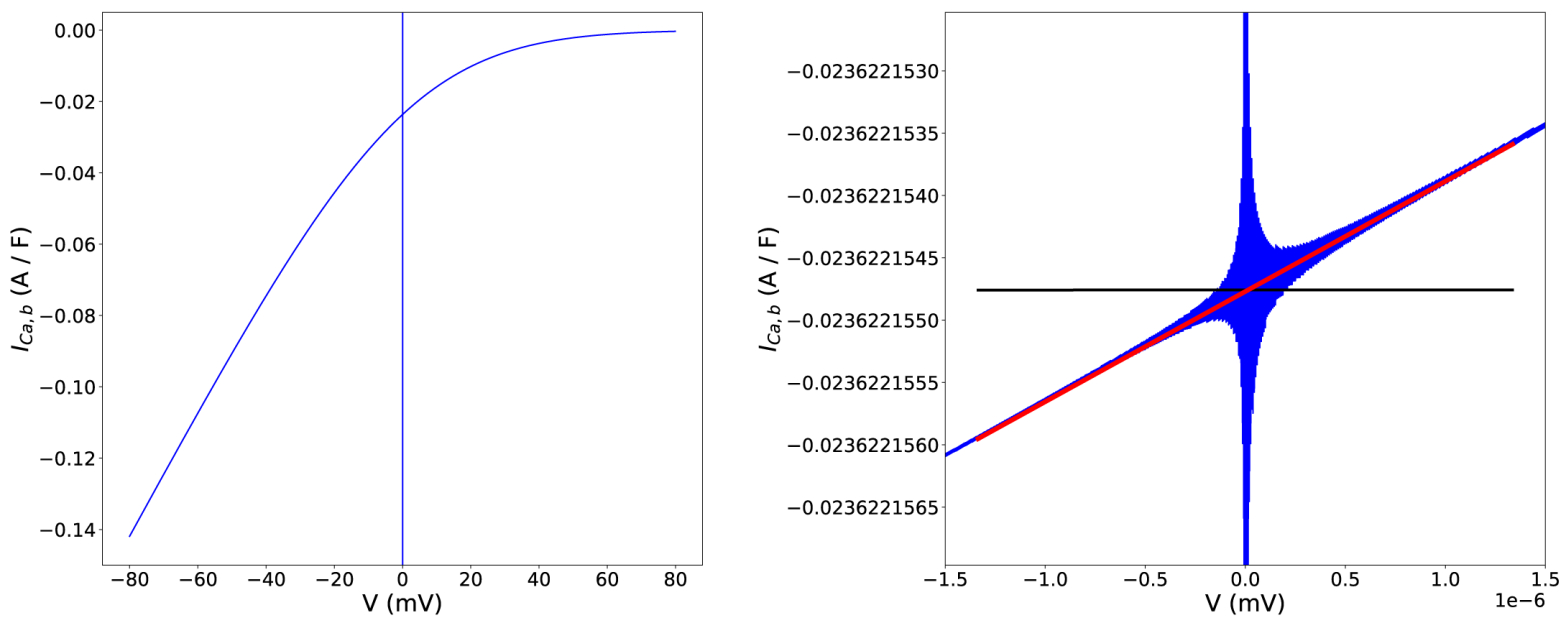
Example singularities fix in the background calcium current ( *I _Ca,b_*) equation of the Davies *et al.* ^17^ model. Left: *I _Ca,b_* as a function of voltage across the physiological range. At, and close to, *V* = 0mV we hit a singularity such that the computation attempts to evaluate 0/0 and answers can tend to ±∞, seen here as apparently vertical lines at *V* = 0 mV. Right: a zoomed-in view around the singularity. The red line represents our applied linear approximation, evaluated in the voltage interval [–1.336 × 10 ^–6^ ≤ *V* ≤ 1.336 × 10 ^–6^] (or equivalently –10 ^–7^ ≤ *U* ≤ + 10 ^–7^). The black line represents a standard fix often manually applied to models, which leads to the discontinuity we observe at *U* = ±10 ^–7^.

As an example, Figure 1 shows the background calcium current *I*_*Ca*,_ *_b_* in the Davies *et al.* ^17^ model of a canine cardiomyocyte, as a function of trans-membrane voltage ( *V*). The figure shows unstable, asymptotically-increasing oscillatory behaviour close to *V* = 0.

By plotting such graphs for a number of cases, Johnstone ^16^ found that the range in which instability occurs numerically is within approximately 10 ^–7^ of *U* = 0 when using double precision (64 bits to represent a floating point number in computer memory).

Problems with these singularities are most apparent when running voltage-clamp experiments, when the voltage *V* is clamped exactly at a singularity voltage *v*_0_. Given the infinite number of choices for voltage clamps and parameters within the models, one might expect this to be unusual. However, this situation is very common. Rounded numbers appear as the clamp voltage in experiments, where steps to a handful of voltages are chosen manually by an experimenter; and also in the model equations themselves. This unfortunate collision is most commonly encountered when the model equations feature *v*_0_ = 0 mV, as in the standard form of the GHK flux equation itself (Equation (11) in Goldman ^15^). But the situation also occurs in the more general form of Equation (1) because parameterisation has commonly been done ‘by hand’ in ion channel modelling, and so the ( *V – v*_0_) terms often feature round numbers for *v*_0_. As an example, equations of the form of Equation (1) with both ( *V* + 10) and ( *V* + 25) appeared in the original Hodgkin and Huxley ^18^ model, meaning that even this fundamental model requires modification to do voltage clamps to –10 or –25 mV (N.B. the voltage in the original paper is defined relative to resting potential rather than extracellular potential, and some CellML implementations have updated these numbers to the modern convention using a similar process to that described in Brown ^19^).

Apart from voltage clamp experiments and singularities at ‘round numbers’, any singularity within normal physiological voltage ranges will be crossed during the simulation of an action potential. Perhaps frustratingly, the more accurately you solve the ODEs, the more likely you are to hit these singularities. This is because a simple fixed-timestep solver is very unlikely to hit the narrow region of voltage which causes a problem, but using an adaptive timestep solver with automatic error correction (like CVODE ^20^) can detect large gradients or sudden changes, introduce more timesteps to refine the solution around these points, ‘home in’ on the singularities and crash. This is definitely not to say that the adaptive solvers are unsuitable for these models, quite the opposite — electrophysiology models frequently have stiff systems with ‘fast sodium current’ upstrokes and other slower processes, and adaptive ODE solvers can provide huge improvements in speed and accuracy. So rather, enabling use of adaptive solvers is an added incentive to avoid the numerical problems associated with singularities.

### Approaches to fix singularities

It is worth noting that the use of computational optimisations such as using the function ‘expm1’ to represent *e ^U^* – 1 do help by increasing precision for small values of *U*, but they do not alleviate the problem entirely.

There are a number of ways the singularity can be removed. When inspecting existing models, we found many CellML files where L’Hôpital’s rule had already been applied manually when numerical problems had been encountered. In the notation we introduced above it is simple to see how L’Hôpital’s rule applies

$$\underset{U\to 0}{\lim}\;g(U)=\underset{U\to 0}{\lim}\;\frac{U}{e^{U}-1}=\underset{U\to 0}{\lim}\;\frac{\frac{\text{d}U}{\text{d}U}}{\frac{\text{d}}{\text{d}U}(e^{U}-1)}=\underset{U\to 0}{\lim}\;\frac{1}{e^{U}}=\frac{1}{1}=1.\;(6)$$

So the ‘fixes’ in most CellML files apply this constant limit value, *g*( *U*) = 1, across a region close to the singularity (with a ‘piecewise’ statement in CellML). Substituting the L’Hôpital limit into Equation (4), this fix is applied as

$$\;C(V)=\{\begin{array}{ll} A(V), & \left|U\right|<\varepsilon ,\\ A(V)\frac{U}{e^{U}-1}, & \text{otherwise}. \end{array}\;(7)$$

Where *∈* is a small range, which varies depending on the CellML file but translates to a small region of voltage. In our notation this is equivalent to |( *V – v*_0_)| > *∈*/ *B*.

A Taylor expansion around the singularity gives $g(U)\approx 1-\frac{U}{2}+\frac{U^{2}}{12}+\ldots$ and so as a refinement of Equation (7), Johnstone ^16^ suggested using the first two terms of the Taylor expansion rather than just the first term, giving a more accurate approximation close to the singularity:

$$\;C(V)=\{\begin{array}{ll} A(V)\left(1-\frac{U}{2}\right), & \left|U\right|<\varepsilon ,\\ A(V)\frac{U}{e^{U}-1}, & \text{otherwise}. \end{array}\;(8)$$

In chaste_codegen, we take this approach with a small modification. Rather than using the above expression we decided to simply ‘draw a line’ between the values of *C*( *V*) evaluated using Equation (1) at the *∈* bounds of our region, and interpolate from this. This was not actually any simpler to implement, as we still identify *U* in order to pick bounds over which to apply the linear approximation. But a benefit of this approach over using Equation (8) is that we get at least *C* ^0^ continuity as we leave the region, even if some curvature in *C*( *V*) is apparent, whereas there can still be (typically very small) discontinuities in *C*( *V*) as we transition between the cases in Equation (8). Note that any discontinuities in Equation (8) are, by definition, much smaller than the discontinuities that appear using Equation (7), as we can see in Figure 1. This continuity can be important to avoid problems in numerical solutions of ODEs, but in practice Equation (8) and the approach we have taken are almost indistinguishable given the size of *∈*.

The algorithm we implemented within cellmlmanip has two parts. To identify whether an equation has a singularity and apply a fix, the algorithm is:

1. Recursively check within each equation for singularities, term by term.
2. Skip (sub-)equations that are piecewise statements, we assume that if these have a singularity it has a manual fix applied.
3. Find U using SymPy’s pattern matching capabilities.
4. Solve for *U* = 0 to find the singularity point in terms of model variables (usually Voltage, *V*).
5. Introduce a piecewise statement to replace the original expression within –10 ^–7^ ≤ *U* ≤ +10 ^–7^ (the fact this is now a nondi- mensional range means that the same fixed range appears to work well for all singularities we have found; it is translated back into voltage ranges of different widths at code generation time).
6. Within this range we use linear interpolation between values evaluated using the original expression at the boundaries ( *U* = *±*10 ^−7^).
7. We note that $\frac{U}{-1+e^{U}}$ leads to a similar situation, as do $\frac{e^{U}-1}{U}$ and $\frac{-1+e^{U}}{U}$ . Therefore these cases are treated similarly.

To ensure we fix the appropriate equation, the algorithm is:

1. A graph is constructed for dependencies of all equations in the model. We can then order them from ODEs at the top level to state variables at the bottom level.
2. All equations in the model are rewritten in terms of state variables, by substituting them into intermediate variables, so that (for instance) *V* appears explicitly in all equations that have any dependence on it.
3. We start at the bottom level of the graph, look for singularities and if none are found using the procedure above we progress to the next level of the graph. If a singularity is found we introduce a fix at that node of the graph.

### Testing

To test the process we searched a set of CellML models (all those annotated for use with Chaste and the WebLab and available at https://github.com/chaste/cellml ^21^) for any piecewise elements. We then manually identified the subset of these being used to fix singularities. We removed these fixes from the CellML files then verified that our code would indeed find and fix these singularities automatically. The result of this exercise is shown in Table 1. The table shows the number of previously hard-coded fixes and the number of extra fixes detected for the range of CellML files. In short, all 106 singularities with previously hard-coded fixes were identified, and the code found 263 new singularities that had never been ‘fixed’ within the CellML files. An overview of all 369 fixes, each with a similar plot to Figure 1, is available in the ‘assets’ branch of the chaste_codegen GitHub page ^22^.

**Table 1.** Comparison of numbers of singularities and hard-coded fixes found in a range of CellML files.

CellML file	# previously hard-coded fixes (all auto-fixed when removed)	# extra detected (all auto- fixed)	total (all auto- fixed)
aslanidi atrial model 2009	0	6	6
aslanidi 2009	0	9	9
beeler reuter model 1977	2	0	2
benson epicardial 2008	0	9	9
bernus wilders zemlin verschelde panfilov 2002 version01	0	1	1
bondarenko 2004 apical	0	1	1
bondarenko 2004 septum	0	1	1
bueno 2007 endo	0	0	0
bueno 2007 epi	0	0	0
Carro Rodriguez Laguna Pueyo CinC2010 ENDO	0	5	5
Carro Rodriguez Laguna Pueyo CinC2010 EPI	0	5	5
clancy rudy 2002	1	5	6
Corrias rabbit purkinje model	3	0	3
courtemanche 1998	7	0	7
davies isap 2012	7	0	7
decker 2009	8	0	8
demir model 1994	0	6	6
difrancesco noble model 1985	5	5	10
dokos model 1996	0	3	3
earm noble model 1990	0	3	3
espinosa model 1998	6	3	9
faber rudy 2000	2	9	11
fink noble giles model 2008	0	1	1
fox model 2001	0	4	4
grandi pasqualini bers 2010	0	6	6
grandi pasqualini bers 2010 endo	0	6	6
hilgemann noble model 1987	4	3	7
hodgkin huxley squid axon model 1952 modified	2	0	2
HundRudy2004 units	0	9	9
iribe model 2006	4	3	7
IyerMazhariWinslow2004	0	4	4
iyer model 2007	0	4	4
jafri rice winslow 1998	0	7	7
kurata model 2002	0	3	3
lindblad atrial model 1996	0	6	6
LivshitzRudy2007	0	8	8
Li Mouse 2010	1	1	2
luo rudy 1991	2	0	2
luo rudy 1994	0	9	9
MahajanShiferaw2008 units	5	0	5
Maleckar	0	1	1
maltsev 2009	3	0	3
matsuoka model 2003	4	0	4
mcallister noble tsien 1975 modelB	0	5	5
noble model 1962	0	3	3
noble model 1991	4	3	7
noble model 1998	4	3	7
noble model 2001	4	6	10
NN SAN model 1984	7	4	11
Noble SAN model 1989	4	4	8
nygren atrial model 1998	0	1	1
ohara rudy 2011 endo	0	5	5
ohara rudy 2011 epi	0	5	5
ohara rudy cipa v1 2017	5	0	5
paci hyttinen aaltosetala severi atrial Version	0	1	1
paci hyttinen aaltosetala severi ventricular Version	0	1	1
pandit clark giles demir 2001 version06 variant01	0	1	1
pandit clark giles demir 2001	0	1	1
pasek simurda christe 2006	0	3	3
pasek model 2008	0	7	7
priebe beuckelmann 1998	1	0	1
ramirez 2000	0	6	6
sachse model 2007	0	1	1
sakmann model 2000 epi	4	6	10
shannon wang puglisi weber bers 2004 model updated	0	10	10
stewart zhang model 2008	0	1	1
tentusscher model 2004 endo	0	1	1
tentusscher model 2004 epi	1	0	1
tentusscher model 2004 M	0	1	1
tentusscher model 2006 endo	1	0	1
tentusscher model 2006 epi	1	0	1
tentusscher model 2006 M	1	0	1
Tomek model13endo	0	8	8
Tomek model13epi	0	8	8
Trovato2020	0	5	5
viswanathan model 1999 epi	2	7	9
wang model 2008	0	3	3
winslow model 1999	1	3	4
zhang SAN model 2000 0D capable	0	4	4
**Total**	**106**	**263**	**369**

There are a number of advantages in removing hard-coded singularity fixes from the CellML files: we can make more accurate fixes around singularities (as shown in Figure 1); others could choose how to treat the singularities and adapt our code to use other methods if they wish; and finally the CellML model is simplified to represent the equations and not how they should be solved. A disadvantage in removing hard-coded singularity fixes from the main CellML repository (PMR) is that all translation code would need to adopt an approach similar to the one we have outlined to avoid hitting singularities, whereas currently around 80 to 90 hard-coded fixes will appear in any generated code without code generation tools needing to do any special treatment. Note that the figure of 106 singularities includes approximately 20 hard-coded fixes we applied to our subset of CellML models using Equation (8) in previous work, which do not feature in the main CellML repository (PMR). Dealing with singularities at code generation time is more future-proof for when people add new models, rather than hard-coding the fixes we applied here into the CellML files in the Physiome Model Repository (PMR) and then having to periodically repeat this exercise for any new models that have been added. On balance, we think that this automated approach at code generation time is the preferable route and hope it has been described so that others can reproduce it in their own code generation software, or re-use components of our open source implementation. In the remainder of this article we discuss the practicalities of using chaste_codegen.

### Operation

There are two main ways to use chaste_codegen. It can be used integrated as part of the Chaste ^5^ build process, from the 2021.1 release onward. It can also be used as a standalone command line tool or Python library. The minimal system requirements for chaste_codegen as a standalone command-line tool are:

• python3 (3.5+), tested on Windows 10, Ubuntu Linux 18.04 and MacOS.
• python3 pip (usually bundled with a python installation).
• python3 venv (or other python virtual environment) is recommended, to ensure the right versions of dependencies are available. However chaste_codegen will still work without a virtual environment. Python3_venv is required for use within Chaste and is usually bundled with a python installation.

For use integrated into the Chaste ^5^ build process, follow the regular guides ^23^ on installing and building Chaste. As part of the Chaste installation process chaste_codegen will be installed in a virtual environment and all CellML files in the source will be converted using the appropriate settings.

To install as a stand-alone command line tool, run pip install chaste_codegen. You may want to create a python virtual environment ( venv) first. The basic usage is: chaste_codegen cellml_file where cellml_file is the CellML file to be converted. To get a detailed overview of the various options run the command chaste_codegen -h.

## Use cases

In this section we will briefly show a number of common conversions in action. For a more detailed guide see the ‘Code generation from CellML’ section in the Chaste guides ^23^. Due to the size of both the import and generated code, however, we will mention only key snippets and refer to the full files available in the assets branch of the chaste_codegen GitHub page ^22^.

In the following examples a CellML 1.0 file for the Hodgkin-Huxley model will be converted into code for a number of different ODE solvers. The CellML file consists of a number of components (membrane, sodium channel, sodium channel m gate, sodium channel h gate, potassium channel, potassium channel n gate and leakage current) and within these components variables are defined. The model also includes and links between components and unit definitions.

CellML files may include metadata through the use of RDF, the Resource Description Framework. chaste_codegen makes use of these annotations when generating C++ source code for Chaste, some of which are optional. In particular, chaste_codegen needs to know which variables represent *voltage* and *stimulus_current*, in order to link the models into the mono/bi-domain equations.

Below an example of a variable called *V* tagged as voltage is shown.

<variable name="V" units =" millivolt " initial_value ="−80" public_interface="out"

                        cmeta : id="membrane_voltage">

     <rdf : RDF xmlns : rdf =" http://www.w3.org/1999/02/22−rdf−syntax−ns#"

                          xmlns : bqbiol="http://biomodels.net/biology−qualifiers/">

         <rdf : Description rdf : about="#membrane_voltage">

           <bqbiol : is

                rdf : resource="https://chaste.comlab.ox.ac.uk/cellml/ns/oxford−metadata#membrane_voltage"/>

         </rdf : Description>

    </rdf : RDF>

</variable>

### ‘Plain’ C++ code

The following command generates what we call ‘Plain’ C++ code. This code is used for solvers that only require the right hand side of the ODE, such as Forward Euler and Runge-Kutta solvers. This kind of code generation does not require any specific flags.

chaste_codegen ModelName.cellml

This generates ModelName.cpp and ModelName.hpp. The code generates a class called CellModelNameFromCellML which inherits from AbstractCardiacCell. It contains the following key methods:

• a constructor and destructor
• UseCellMLDefaultStimulus calculates a stimulus based on parameters (amplitude, duration, start- and end-time) set in the model. These are identified using metadata as described above.
• GetIIonic calculates total ionic current at the present time (for use in tissue simulations).
• EvaluateYDerivatives calculates the derivatives of the the state values when provided with their current values, defining the ODEs of the model.
• ComputeDerivedQuantities gives a way to calculate the value of quantities that are derived directly from state variables, for example currents such as “the fast sodium current”.
• OdeSystemInformation::Initialise gives a way to retrieve information about the model such as name, free variable (usually time), state variable, modifiable parameters and named derived quantities.

### CVODE

The following command generates code for the CVODE solver which has its own vector class.

chaste_codegen –cvode –use-analytic-jacobian ModelName.cellml

This generates .cpp and .hpp files with the same name as before. The generated class now inherits from AbstractCvodeCell, containing the same methods but with SUNDIALS’ vector class for use directly with CVODE. It also has a method EvaluateAnalyticJacobian in which the analytic Jacobian is defined, to be used by CVODE.

### Backward Euler

The following command generates Backward Euler code.

chaste_codegen –backward-euler ModelName.cellml

This generates .cpp and .hpp files with the same name as before. The generated class inherits from AbstractBackwardEulerCardiacCell. The class does not have EvaluateYDerivatives, but instead it has:

• UpdateTransmembranePotential where the voltage is updated based on the ODE for voltage.
• ComputeOneStepExceptVoltage where the other state variables are updated. The variables are described by linear equations use a backward Euler fashion e.g. *n* = ( *n* + ( *α* ∗ *mDt*)) */* (1.0 – ((– *α* – *β*) ∗ *mDt*))

### Rush-Larsen

The following command generates code in which some state variables are updated using analytic solutions using the Rush-Larsen scheme ^24^.

chaste_codegen –rush-larsen ModelName.cellml

This generates .cpp and .hpp files with the same name as before. The generated class now inherits from AbstractRushLarsenCardiacCell. The concrete class does not contain a method EvaluateYDerivatives, but instead it has methods:

• EvaluateEquations where the voltage and non-linear state-variables are updated using the Forward Euler method. For the linear equation state variables variables are stored to capture the analytic solution.
• ComputeOneStepExceptVoltage where the linear state-variables are updated using their analytic solutions.

## Summary

This paper has introduced the software tool chaste_codegen, designed to translate electrophysiology models from the CellML XML format into C++ code for use by the Chaste simulation package. We have shown how the tool can be used, highlighted the main different types of code it can generate for different solvers and shown a number of advanced features that chaste_codegen implements over a previous tool called PyCML. The most notable are the ability to generate analytic Jacobians and to evaluate and fix singularities in equations. We have shown that the singularity analysis works as expected with an analysis of a large number of popular models, by identifying all previously-identified singularities and finding over three times as many in total.

## Contributing to development

chaste_codegen and the cellmlmanip libraries are open-source and publicly available and we welcome contributions: from questions about the current functionality to suggestions for improvements and source code contributions. It should also be relatively simple to extend the use of templates to generate code for other simulation packages in C++, Python, or other languages. Contributions are made in the first instance using GitHub issues. In order to contribute, users create a new issue in either GitHub repository or comment on an existing issue. Contributions in the form of source code can be made by issuing a pull request on either repository (ideally a new GitHub issue should be created, which can then be linked to the pull-request). The pull request will trigger an automated test suite and a number of other checks for things such as code formatting and test coverage.

## Software availability

Software available from: https://pypi.org/project/chaste-codegen/

Source code available from: https://github.com/ModellingWebLab/chaste-codegen

Archived source code as at time of publication: https://doi.org/10.5281/zenodo.5527756

BSD 3-Clause License

## References

[1] Garny A Nickerson DP Cooper J : CellML and associated tools and techniques. *Philos Trans A Math Phys Eng Sci.* 2008;366(1878):3017–3043. 10.1098/rsta.2008.0094 18579471

[2] Sarwar DM Kalbasi R Gennari JH : Model annotation and discovery with the physiome model repository. *BMC Bioinformatics.* 2019;20(1):457. 10.1186/s12859-019-2987-y 31492098 PMC6731580

[3] Yu T Lloyd CM Nickerson DP : The physiome model repository 2. *Bioinformatics.* 2011;27(5):743–744. 10.1093/bioinformatics/btq723 21216774

[4] Clerx M Cooling MT Cooper J : CellML 2.0. *J Integr Bioinform.* 2020;17(2–3):20200021. 10.1515/jib-2020-0021 32759406 PMC7756617

[5] Cooper FR Baker RE Bernabeu MO : Chaste: Cancer, heart and soft tissue environment. * J Open Source Softw.* 2020;5(47):1848. 10.21105/joss.01848 PMC7614534 37192932

[6] Garny A Hunter PJ : Opencor: a modular and interoperable approach to computational biology. *Front Physiol.* 2015;6:26. 10.3389/fphys.2015.00026 25705192 PMC4319394

[7] Clerx M Collins P de Lange E : Myokit: a simple interface to cardiac cellular electrophysiology. *Prog Biophys Mol Biol.* 2016;120(1–3):100–114. 10.1016/j.pbiomolbio.2015.12.008 26721671

[8] Cooper J Scharm M Mirams GR : The cardiac electrophysiology web lab. *Biophys J.* 2016;110(2):292–300. 10.1016/j.bpj.2015.12.012 26789753 PMC4724653

[9] Daly AC Clerx M Beattie KA : Reproducible model development in the cardiac electrophysiology Web Lab. *Prog Biophys Mol Biol.* 2018;139:3–14. 10.1016/j.pbiomolbio.2018.05.011 29842853 PMC6288479

[10] On the application of partial evaluation to the optimisation of cardiac electrophysiological simulations, PEPM ’ 06, New York, NY USA, Association for Computing Machinery.2006. 10.1145/1111542.1111546

[11] Jinja: Jinja Documentation (3.0.x). Reference Source

[12] Meurer A Smith CP Paprocki M : Sympy: symbolic computing in python. *PeerJ Comput Sci.* 2017;3:e103. 10.7717/peerj-cs.103

[13] Cooper J Spiteri RJ Mirams GR : Cellular cardiac electrophysiology modeling with Chaste and CellML. *Front Physiol.* Publisher: Frontiers,2015;5:511. 10.3389/fphys.2014.00511 25610400 PMC4285015

[14] Hindmarsh AC Brown PN Grant KE : Sundials: Suite of nonlinear and differential/algebraic equation solvers. *ACM Transactions on Mathematical Software (TOMS).* 2005;31(3):363–396. 10.1145/1089014.1089020

[15] Goldman DE : Potential, impedance, and rectification in membranes. *J Gen Physiol.* 1943;27(1):37–60. 10.1085/jgp.27.1.37 19873371 PMC2142582

[16] Johnstone RH : Uncertainty characterisation in action potential modelling for cardiac drug safety.PhD Thesis, University of Oxford,2018. Reference Source

[17] Davies MR Mistry HB Hussein L : An in silico canine cardiac midmyocardial action potential duration model as a tool for early drug safety assessment. *Am J Physiol Heart Circ Physiol.* 2012;302(7):H1466–H1480. 10.1152/ajpheart.00808.2011 22198175

[18] Hodgkin AL Huxley AF : A quantitative description of membrane current and its application to conduction and excitation in nerve. *J Physiol.* 1952;117(4):500–544. 10.1113/jphysiol.1952.sp004764 12991237 PMC1392413

[19] Brown AM : The classics updated, or an act of electrophysiological sacrilege? *J Physiol.* 2019;597(11):2821–2825. 10.1113/JP276771 31148193

[20] Cohen SD Hindmarsh AC Dubois PF : CVODE, A Stiff/Nonstiff ODE Solver in C. *Computers in Physics.* 1996;10(2):138. 10.1063/1.4822377

[21] Chaste/cellml.2021. Reference Source

[22] ModellingWebLab/chaste-codegen. Reference Source

[23] ChasteGuides.Chaste. Reference Source

[24] Rush S Larsen H : A practical algorithm for solving dynamic membrane equations. *IEEE Trans Biomed Eng.* 1978;25(4):389–392. 10.1109/TBME.1978.326270 689699

