# Supplementary material for: cellmlmanip and chaste_codegen: automatic CellML to C++ code generation with fixes for singularities and automatically generated Jacobians
Source: Wellcome Open Res. 2022 Jun 15;6:261. Originally published 2021 Oct 12. [Version 2] doi: 10.12688/wellcomeopenres.17206.2 (PMC8902258; doi:10.12688/wellcomeopenres.17206.2)
Supplement: Peer review history

# Peer review: Reviewer response for version 2

**DOI:** 10.21956/wellcomeopenres.19920.r51088

**Published:** 2022-06-29

**Authors:** David Nickerson

**Affiliations:** 1 Auckland Bioengineering Institute, University of Auckland, Auckland, New Zealand

Many thanks for the detailed replies and manuscript updates!

Are the conclusions about the tool and its performance adequately supported by the findings presented in the article?

Yes

Is the rationale for developing the new software tool clearly explained?

Yes

Is the description of the software tool technically sound?

Yes

Are sufficient details of the code, methods and analysis (if applicable) provided to allow replication of the software development and its use by others?

Yes

Is sufficient information provided to allow interpretation of the expected output datasets and any results generated using the tool?

Yes

Reviewer Expertise:

Standards development, cardiac electrophysiology modelling, software development.

I confirm that I have read this submission and believe that I have an appropriate level of expertise to confirm that it is of an acceptable scientific standard.

# Peer review: Reviewer response for version 2

**DOI:** 10.21956/wellcomeopenres.19920.r51086

**Published:** 2022-06-17

**Authors:** Axel Loewe

**Affiliations:** 1 Institute of Biomedical Engineering (IBT), Karlsruhe Institute of Technology (KIT), Karlsruhe, Germany

Thank you for the clarifications and improvements.

Are the conclusions about the tool and its performance adequately supported by the findings presented in the article?

Yes

Is the rationale for developing the new software tool clearly explained?

Yes

Is the description of the software tool technically sound?

Yes

Are sufficient details of the code, methods and analysis (if applicable) provided to allow replication of the software development and its use by others?

Yes

Is sufficient information provided to allow interpretation of the expected output datasets and any results generated using the tool?

Yes

Reviewer Expertise:

Computational cardiac modeling

I confirm that I have read this submission and believe that I have an appropriate level of expertise to confirm that it is of an acceptable scientific standard.

# Peer review: Reviewer response for version 1

**DOI:** 10.21956/wellcomeopenres.19013.r48329

**Published:** 2022-03-07

**Authors:** Rahuman S Malik-Sheriff

**Affiliations:** 1 European Molecular Biology Laboratory, European Bioinformatics Institute (EMBL-EBI), Wellcome Genome Campus, Hinxton, Cambridgeshire, UK

This article describes the chaste_codegen tool developed to convert a CellML model to C++ code. chaste_codegen is a Python3 implementation of the previously used Python 2 based PyCML tool. The authors describe in detail two new additional features implemented in chaste_codegen (1) Jacobian matrix generation (2) the ability to automatically detect and fix singularities in the equation with linear approximation. And authors clearly demonstrate the validity of their solution to detect and fix singularities with several examples. chaste_codegen along with the new features is a quite useful tool.

 The article is well written, however, it could benefit the readers further if the following minor suggestions are considered.

  

1. It would be useful to provide a short summary of the major functionalities of chaste_codegen in the introduction. Although one would expect them to be similar to PyCML, it would useful to briefly describe them for the benefit of the new users.
2. Also, it would be useful to discuss the performance of chaste_codegen when compared against PyCML. For example, it will be useful to briefly mention how well does the computational time compares in general. Were the functionalities from PyCML comparable to those in chaste_codegen  excluding the new features (automated Jacobians and singularity fixing)?
3. The article describes in detail the rationale and solution to fix singularities in the equations (where the value of variable such as Voltage tends to become 0/0).  The chaste_codegen has been already integrated into chaste. Authors could discuss how this tool or approach could be used beyond chaste and provide a few suggestions.
4. I tried to run and test the standalone chaste_codegen python tool. Although I tried only for an hour or so, I couldn’t get it working. I managed to get it installed in a virtual environment as suggested, but couldn’t export the cpp file from the CellML model. It will be useful to provide some sort of “test_codegen” command to run sample tests and report errors to help to troubleshoot.
5. A concern on the tool is that it is based on cellmlmanip which supports only CellML 1.0 and the author described there are no plans to support CellML 2.0. Would this mean the chaste_codegen will be outdated when CellML 2.0 is adapted widely? It would be useful to discuss this in the article, as a part of the limitation if so. Also, would chaste_codegen throw a clear warning if CellML 2.0 is used?
6. I would suggest authors, to thoroughly check all the equations (1 - 8) and ensure all components are properly defined.  For example, *V0 * is defined as the voltage at which we hit the singularity, but there is no definition for B in equation 1. Although, it might be trivial to those working in electrophysiology modeling and references are provided, it will be useful to describe functions such as C(V), A(V) in the text to help general readers.
7. Validity of the singularity detection by chaste_codegen was shown through the comparison of the number of singularities detected and the hardcoded fixes in previous CellML models (Table 1). Similarly, the authors described that the validity of the generated Jacobians was assessed by comparing numerical results of runs of a number of different models without providing further details. If not in detail, at least a few sentences on how many models were used and so on would be beneficial.
8. The authors have discussed the advantages of the tool and the newly developed features, it would be useful to also discuss limitations if any.
9. It will be also useful if the authors discuss any comparable existing approaches and implementation for automatic singularities detection and fixing.

Are the conclusions about the tool and its performance adequately supported by the findings presented in the article?

Yes

Is the rationale for developing the new software tool clearly explained?

Yes

Is the description of the software tool technically sound?

Yes

Are sufficient details of the code, methods and analysis (if applicable) provided to allow replication of the software development and its use by others?

Yes

Is sufficient information provided to allow interpretation of the expected output datasets and any results generated using the tool?

Yes

Reviewer Expertise:

I am a mathematical modeler and one of the editorial board members of the community standard, SBML. I am familiar with XML-based formats to describe models and the libraries developed to handle them. I also lead the BioModels, a repository of mathematical models of biological systems.

I confirm that I have read this submission and believe that I have an appropriate level of expertise to confirm that it is of an acceptable scientific standard.

# Response

**Published:** 2022-06-08

**Authors:** Gary Mirams

**Affiliations:** University of Nottingham, UK

*It would be useful to provide a short summary of the major functionalities of chaste_codegen in the introduction. Although one would expect them to be similar to PyCML, it would useful to briefly describe them for the benefit of the new users.*

 We have expanded the introduction to point out the principal features of PyCML which indeed chaste_codegen also provides. 

*Also, it would be useful to discuss the performance of chaste_codegen when compared against PyCML. For example, it will be useful to briefly mention how well does the computational time compares in general. Were the functionalities from PyCML comparable to those in chaste_codegen  excluding the new features (automated Jacobians and singularity fixing)?*

 We haven’t done a thorough comparison of performance other than that it was fast enough and did not noticably impact the CellML conversion time during Chaste compilation (in either direction!). As the reviewer has pointed out, chaste_codegen has a number of features PyCML misses. The primary concern for users is almost certainly run time of the generated code (solving ODEs) rather than this code generation step. 

*The article describes in detail the rationale and solution to fix singularities in the equations (where the value of variable such as Voltage tends to become 0/0).  The chaste_codegen has been already integrated into chaste. Authors could discuss how this tool or approach could be used beyond chaste and provide a few suggestions.*

 We have made some suggestions about using Jinja2 templates and pointed to their documentation. 

*I tried to run and test the standalone chaste_codegen python tool. Although I tried only for an hour or so, I couldn’t get it working. I managed to get it installed in a virtual environment as suggested, but couldn’t export the cpp file from the CellML model. It will be useful to provide some sort of “test_codegen” command to run sample tests and report errors to help to troubleshoot.*

 Running the command chaste_codegen –h gives detailed help. Having said that, chaste_codegen should convert it.  Chaste_codegen has been tested against all the models in https://github.com/Chaste/cellml It would be interesting to know what errors the reviewer faced, and we would encourage them to leave an issue on the github repository.  We do require the models to have metadata annotation which is not present in most models in the CellML repository. We have highlighted this in the paper. 

*A concern on the tool is that it is based on cellmlmanip which supports only CellML 1.0 and the author described there are no plans to support CellML 2.0. Would this mean the chaste_codegen will be outdated when CellML 2.0 is adapted widely? It would be useful to discuss this in the article, as a part of the limitation if so. Also, would chaste_codegen throw a clear warning if CellML 2.0 is used?*

 The plan here has been that libCellml would replace cellmlmanip once it has been developed enough for us to use. This would bring with it CellML 2.0 support.  Cellmlmanip is not able to recognise if an XML file is in fact a CellML 2.0 file. Therefore currently attempts to convert a CellML 2.0 file would lead to error messages related to the XML schema validation, informing the user that the file is not valid CellML (1.0). 

*I would suggest authors, to thoroughly check all the equations (1 - 8) and ensure all components are properly defined.  For example, V0  is defined as the voltage at which we hit the singularity, but there is no definition for B in equation 1. Although, it might be trivial to those working in electrophysiology modeling and references are provided, it will be useful to describe functions such as C(V), A(V) in the text to help general readers.*

 These were generic functions or constants that could take any form to fit particular models, we have attempted to clarify this in the text. 

*Validity of the singularity detection by chaste_codegen was shown through the comparison of the number of singularities detected and the hardcoded fixes in previous CellML models (Table 1). Similarly, the authors described that the validity of the generated Jacobians was assessed by comparing numerical results of runs of a number of different models without providing further details. If not in detail, at least a few sentences on how many models were used and so on would be beneficial.*

 We added the number of tests and explained the results in more detail. 

*The authors have discussed the advantages of the tool and the newly developed features, it would be useful to also discuss limitations if any.*

 We have added a few sentences on limitations, mainly in terms of the singularity detection only working for the GHK-flux style equations we discuss here. 

**

*It will be also useful if the authors discuss any comparable existing approaches and implementation for automatic singularities detection and fixing.*

 We have not seen any comparable implementations for automatic singularity fixing, all existing approaches have relied on spotting these and fixing manually as far as we are aware.

# Peer review: Reviewer response for version 1

**DOI:** 10.21956/wellcomeopenres.19013.r48112

**Published:** 2022-03-01

**Authors:** David Nickerson

**Affiliations:** 1 Auckland Bioengineering Institute, University of Auckland, Auckland, New Zealand

This article very nicely lays out the rationale for the chaste_codegen tool, particularly in the evolution from the previous PyCML software used in Chaste. While clearly the emphasis is on producing code suitable for use in Chaste, the authors do suggest how the tool could be used independently. Some introduction or pointers as to how readers might be able to edit/alter the Jinja2 templates to produce non-Chaste code would be helpful.

 While the performance and correctness of the analytic Jacobians generated by chaste_codegen using SymPy is very briefly mentioned, seeing some of those checks would I think be interesting to readers.

 A large portion of this manuscript is describing the use of cellmlmanip to detect and fix singularities. While this is a valuable contribution the authors preface this by suggesting the replacement of cellmlmanip with the libCellML when looking at handling more recent versions of the CellML specification. Perhaps a bit more clarity regarding the future use of cellmlmanip and chaste_codegen regarding the singularity detection and fixing would be helpful.

 I needed the assistance of google to learn what expm1 is, so perhaps other readers might appreciate a pointer.

 Step 2 in the algorithm to identify singularities in equations seems a bit too general. While historically most cardiac electrophysiological models have been encoded in CellML in a similar manner for which this assumption holds true, it is not likely to always be true. Particularly if readers were looking to build on chaste_codegen to work with models from other domains.

 The discussion regarding the removal of hard-coded singularity fixes from the original models available in the main CellML repository is great to see. And something that the authors should be encouraged to discuss with the broader CellML community.

 (Minor comment: `chaste_codegen -h` suggests that the cellml_file could be a URI, but pointing to, for example, https://raw.githubusercontent.com/Chaste/cellml/master/cellml/hodgkin_huxley_squid_axon_model_1952_modified.cellml fails with a file not found error.)

Are the conclusions about the tool and its performance adequately supported by the findings presented in the article?

Yes

Is the rationale for developing the new software tool clearly explained?

Yes

Is the description of the software tool technically sound?

Yes

Are sufficient details of the code, methods and analysis (if applicable) provided to allow replication of the software development and its use by others?

Yes

Is sufficient information provided to allow interpretation of the expected output datasets and any results generated using the tool?

Yes

Reviewer Expertise:

Standards development, cardiac electrophysiology modelling, software development.

I confirm that I have read this submission and believe that I have an appropriate level of expertise to confirm that it is of an acceptable scientific standard.

# Response

**Published:** 2022-06-08

**Authors:** Gary Mirams

**Affiliations:** University of Nottingham, UK

*This article very nicely lays out the rationale for the chaste_codegen tool, particularly in the evolution from the previous PyCML software used in Chaste. While clearly the emphasis is on producing code suitable for use in Chaste, the authors do suggest how the tool could be used independently. Some introduction or pointers as to how readers might be able to edit/alter the Jinja2 templates to produce non-Chaste code would be helpful.*

 This is an interesting thought, and indeed we have added the ability to generate labview and more generic C code since submitting this article. We added a brief discussion of this to the paper. 

*While the performance and correctness of the analytic Jacobians generated by chaste_codegen using SymPy is very briefly mentioned, seeing some of those checks would I think be interesting to readers.*

 The resulting Jacobians from Maple and from sympy look quite different. For a few simple toy examples we manually verified them, but beyond that, the verification is based on the converted models using the sympy generated Jacobians passing the same set of numeric tests against reference results as we previously ran for the Maple-generated Jacobians.  Some further information about numbers of models tested has been added. 

*A large portion of this manuscript is describing the use of cellmlmanip to detect and fix singularities. While this is a valuable contribution the authors preface this by suggesting the replacement of cellmlmanip with the libCellML when looking at handling more recent versions of the CellML specification. Perhaps a bit more clarity regarding the future use of cellmlmanip and chaste_codegen regarding the singularity detection and fixing would be helpful.*

 While this functionality is indeed in located cellmlmanip at the moment, it would be quite straightforward to move to chaste_codegen, or elsewhere such as a future version of libCellML. It does not rely in cellmlmanip specifically, but just requires an algebraic representation of the equations such as SymPy uses. We added text to explain this in the Implementation section. 

*I needed the assistance of google to learn what expm1 is, so perhaps other readers might appreciate a pointer.*

 We add an explanation in brackets when it is first encountered. 

*Step 2 in the algorithm to identify singularities in equations seems a bit too general. While historically most cardiac electrophysiological models have been encoded in CellML in a similar manner for which this assumption holds true, it is not likely to always be true. Particularly if readers were looking to build on chaste_codegen to work with models from other domains.*

 It is indeed a limitation that we will only spot these GHK-flux style equations. One nice feature is that we still spot them even when intermediate equations are involved with our recursive approach. It would be possible to do further ‘pattern matching’ approaches on a case-by-case basis, or to use SymPy’s inbuilt methods ( https://docs.sympy.org/latest/modules/calculus/index.html#sympy.calculus.singularities.singularities ) to find singularities in almost any expression, we tried this initially but found it was a lot slower than our approach (minutes rather than less than a second for a moderately large action potential model, and we even found some examples where the method didn’t terminate at all). 

*The discussion regarding the removal of hard-coded singularity fixes from the original models available in the main CellML repository is great to see. And something that the authors should be encouraged to discuss with the broader CellML community.*

 Thanks for that suggestion, we will be attending CellML workshops and COMBINE etc. to discuss these issues. 

*Minor comment on URIs*

 Thank you for pointing this out! It is indeed the case that we decided against implementing the ability to convert a remote URI, we updated the help text to reflect this in the current release of chaste_codegen.

# Peer review: Reviewer response for version 1

**DOI:** 10.21956/wellcomeopenres.19013.r48330

**Published:** 2022-02-08

**Authors:** Axel Loewe

**Affiliations:** 1 Institute of Biomedical Engineering (IBT), Karlsruhe Institute of Technology (KIT), Karlsruhe, Germany

Hendrix et al. present *chaste_codegen*, a Python 3 reimplementation of the *PyCML* software package which adds new functionality, mostly through integration of the *cellmlmanip* library. Flexible code generation for CellML models is a relevant need in the cardiac modeling community and the tool is well presented in general.

**A few remarks:**

- It seems that most of the new functionality (fixes for singularities) comes directly through cellmlmanip: https://github.com/ModellingWebLab/cellmlmanip/. As such, it might be helpful to explain the relation between cellmlmanip and chaste_codegen early in the manuscript. In the current form it seems a bit odd that half of the 12 text pages of the manuscript deal not with chaste_codegen but with one of its dependencies.
- It might be helpful for the reader to point out the main differences between chaste_codegen and other tools providing similar functionality like for example Myokit (partly from the same authors)
- In general, the tool could be valuable for developers (and potentially also users) of other cardiac electrophysiology simulators. To increase the value beyond the Chaste user community, it would be helpful to outline how the Jinja2 templates would need to be adapted or extended. For example Introduction in the (second paragraph, first sentence) and Methods (fourth paragraph, second-last sentence) sections.
- Why are specific operating systems mentioned? I would have assumed that chaste_codegen runs on all platforms that provide a Python interpreter.

**Typos and other trivia:**

- Title: consider adding the dash in "chaste_codegen" 
- Abstract: consider "new cellmlmanip Python library" -> "cellmlmanip Python library"
- Introduction:
  - consider "hundreds of models" -> "hundreds of mathematical models"
  - The first sentence of the second paragraph is a bit confusing. Why is chaste_codegen needed if CellML models can be imported in Chaste already? Or is this only the case with PyCML/chaste_codegen?
- Methods:
  - Please double check appropriate references to software mentioned in the manuscript (cellmlmanip, SymPy etc.)
  - CellMl -> CellML
  - Cellmlmanip is typed with capital c and in normal font in the second paragraph
  - RDF should be spelled out when it is first mentioned
  - Second paragraph, last sentence: is an "and" missing here?
  - Often, the Goldman–Hodgkin–Katz voltage equation is referred to as "the GHK equation". Here, you refer to the GHK flux equation. Mentioning this explicitly can help to avoid confusion. 
  - p.5: some readers might not be familiar with "expm1", please provide some more background
  - p.6, point 5: hyphenation in nondi-mensional
  - Table 1: model naming (w, w/o "model" etc.) and capitalization appear arbitrary
- Operation:
  - "can be used integrated" -> "can be used"?
  - p.12: "pull-request" -> "pull request" for the sake of consistency

Are the conclusions about the tool and its performance adequately supported by the findings presented in the article?

Yes

Is the rationale for developing the new software tool clearly explained?

Yes

Is the description of the software tool technically sound?

Yes

Are sufficient details of the code, methods and analysis (if applicable) provided to allow replication of the software development and its use by others?

Yes

Is sufficient information provided to allow interpretation of the expected output datasets and any results generated using the tool?

Yes

Reviewer Expertise:

Computational cardiac modeling

I confirm that I have read this submission and believe that I have an appropriate level of expertise to confirm that it is of an acceptable scientific standard, however I have significant reservations, as outlined above.

# Response

**Published:** 2022-06-08

**Authors:** Gary Mirams

**Affiliations:** University of Nottingham, UK

*It seems that most of the new functionality (fixes for singularities) comes directly through cellmlmanip: https://github.com/ModellingWebLab/cellmlmanip/. As such, it might be helpful to explain the relation between cellmlmanip and chaste_codegen early in the manuscript. In the current form it seems a bit odd that half of the 12 text pages of the manuscript deal not with chaste_codegen but with one of its dependencies. This is a fair point. Cellmlmanip is developed by us to parse CellML into sets of equations, and is also used in other projects. Chaste_codegen then takes care of the described manipulations.*

 We have altered the title to reflect that we are introducing both new tools, and clarified their roles in the Introduction section of the paper and the start of the Methods. 

*It might be helpful for the reader to point out the main differences between chaste_codegen and other tools providing similar functionality like for example Myokit (partly from the same authors) The biggest difference is that chaste_codegen is specifically designed to generate (C++) code that for chaste, so that it can be compiled and used in a chaste simulation. Myokit for example is more general purpose.*

 We pointed this out better in the introduction that the main job of chaste_codegen is to generate code for chaste. 

*In general, the tool could be valuable for developers (and potentially also users) of other cardiac electrophysiology simulators. To increase the value beyond the Chaste user community, it would be helpful to outline how the Jinja2 templates would need to be adapted or extended. For example Introduction in the (second paragraph, first sentence) and Methods (fourth paragraph, second-last sentence) sections.*

  This is an interesting thought, and indeed we have added the ability to generate LabView and more generic C code since submitting this article. We added a brief discussion of this to the paper. 

*Why are specific operating systems mentioned? I would have assumed that chaste_codegen runs on all platforms that provide a Python interpreter.*

  We have clarified that these are the platforms we have tested the software on. While Python in theory is platform independent, in practice there are subtle difference how things like file paths work as well as not all libraries are available for all operating systems. While there is no obvious reason to assume that it does not work on other platforms we can also not guarantee that it does. 

*Typos and other trivia:*  We thank the reviewer for these remarks and have corrected the typos and minor comments
